# Supplementary material for: Unmasking GluN1/GluN3A excitatory glycine NMDA receptors
Source: Nat Commun. 2018 Nov 13;9:4769. doi: 10.1038/s41467-018-07236-4 (PMC6233196; doi:10.1038/s41467-018-07236-4)
Supplement: Supplementary file 1 — Supplementary Information [file 41467_2018_7236_MOESM1_ESM.pdf]

Unmasking GluN1/GluN3A excitatory glycine NMDA receptors

Teddy Grand, Sarah Abi Gerges, Mélissa David, Marco A. Diana, Pierre Paoletti

Contents:

Supplementary Figures 1-3

Supplementary References

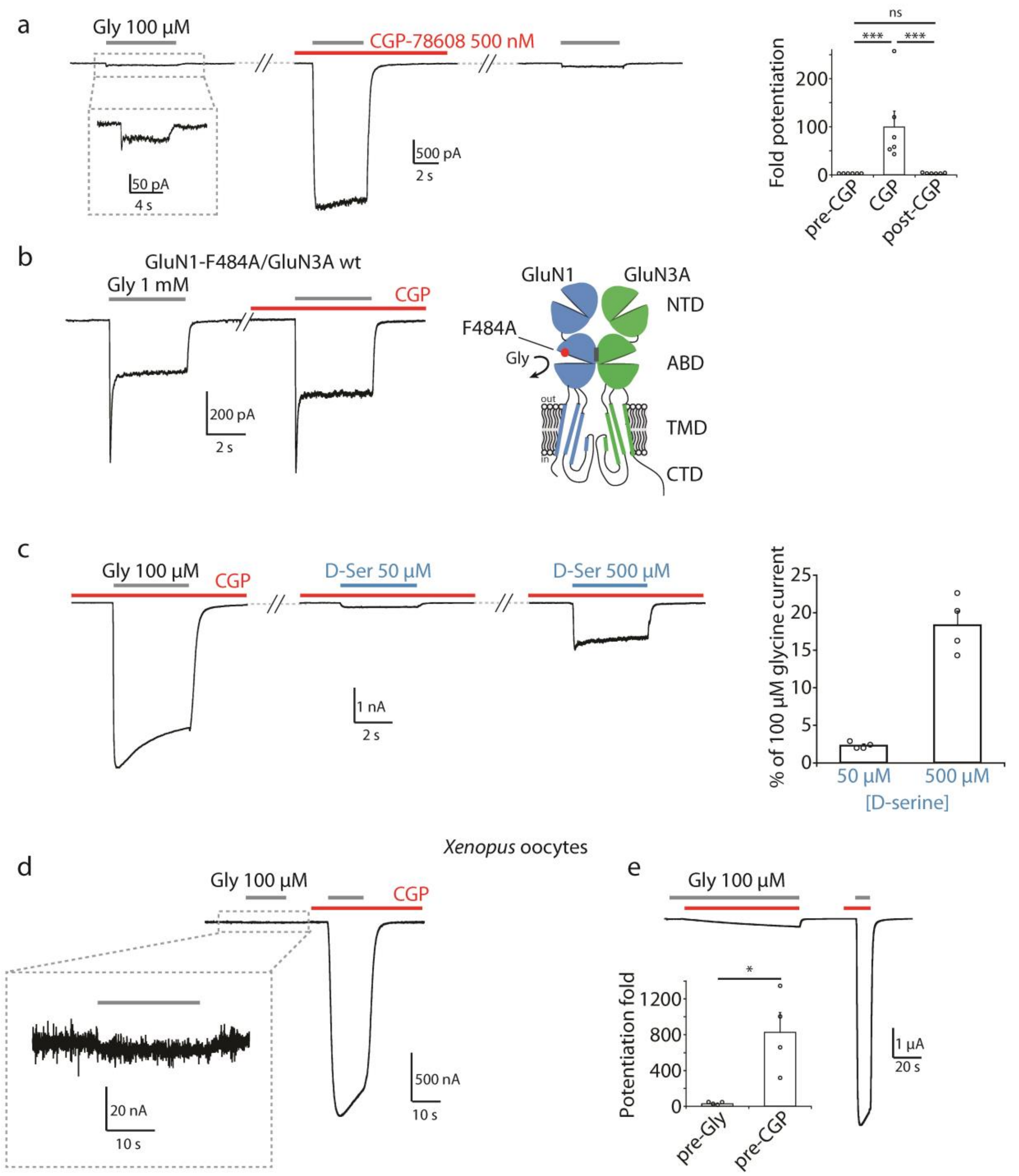

Supplementary Figure 1: Properties of CGP-78608 potentiation

(a) CGP-78608 potentiation is fully reversible. Recording were performed on the same cell. n.s. P=1.0; *** P<0.001; Student’s *t*-test (n=6).

(b) GluN1/GluN3A receptors harboring glycine binding disabled GluN1 subunits (GluN1-F484A mutation) are insensitive to CGP-78608 (500 nM). The fast desensitization of current responses observed upon glycine application likely stems from residual binding of glycine on mutant GluN1 ABD (see ref 1).

(c) D-serine has lower efficacy than glycine at GluN1/GluN3A receptors. CGP-78608 is applied at 500 nM. Bar graph (n=4).

(d) Pre-application of CGP-78608 (500 nM) massively potentiates excitatory glycine GluN1/GluN3A responses recorded in *Xenopus* oocytes.

(e) CGP-78608 potentiation is state-dependent. Potentiation by CGP-78608 (500 nM) is greatly reduced when the compound is applied after, rather than before, application of glycine (pre-Gly *vs* post-Gly, respectively). Bar graph: potentiation measured at steady-state. * P=0.029, Mann-Whitney (n=4).

Recordings from HEK cells (panels a to c) or *Xenopus* oocytes (d and e).

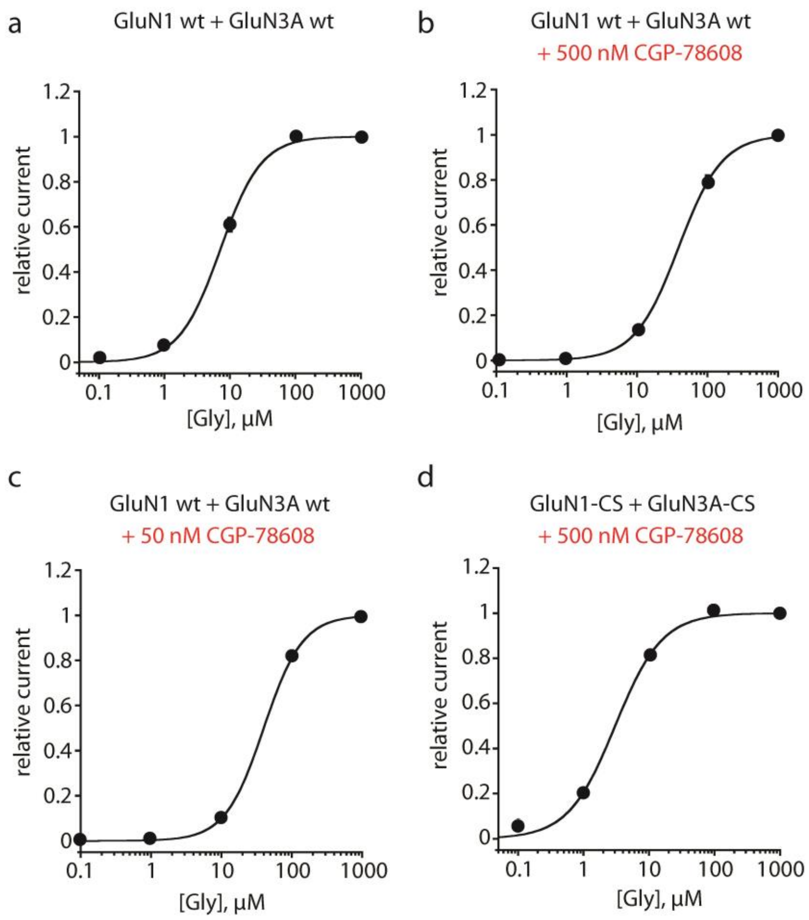

Supplementary Figure 2: Glycine sensitivity of wild-type and redox mutant GluN1/GluN3A receptors.

(a) Glycine concentration-response curve obtained for wild-type (wt) receptors. EC_{50} = 7.1 ± 0.4 µM, n_H = 1.39 (n=5-15).

(b) Glycine concentration-response curve for wild-type receptors in presence of 500 nM CGP-78608. EC_{50} = 38.9 ± 0.8 µM, n_H = 1.47 (n=6).

(c) Same as in (b) but in presence of 50 nM CGP-78608. EC_{50} = 40 ± 0.8 µM, n_H = 1.58 (n=9).

(d) Glycine concentration-response curve for GluN1-CS/GluN3A-CS receptors in the presence of 500 nM CGP-78608. EC_{50} = 3.0 ± 0.3 µM, n_H = 1.21 (n=6).

All recordings were performed in *Xenopus* oocytes and currents measured at the peak.

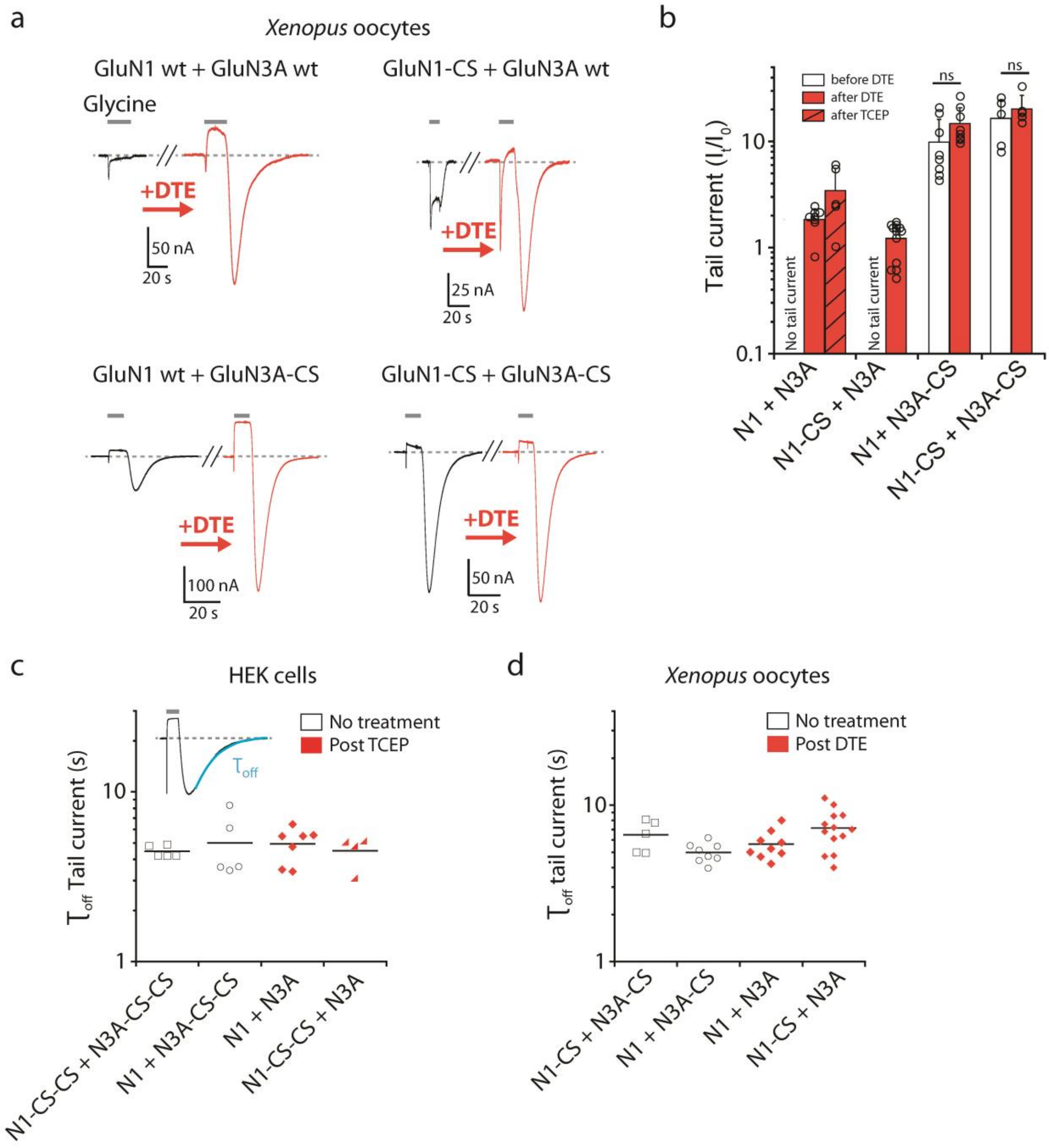

Supplementary Figure 3: Tail current kinetics of receptors lacking the redox-sensitive GluN3A ABD disulfide bridge

(a) Effect of DTE treatment (5 mM, 20 min) on wild-type (wt) mutant GluN1/GluN3A receptors expressed in *Xenopus* oocytes. Each pair of current traces corresponds to responses before and after DTE treatment on the same cell. Glycine was applied at 100 µM.

(b) Quantification of tail currents as described in Figure 2c. n.s. P=0.105, Mann-Whitney and P=0.453, Student's *t*-test respectively (n=5-13).

(c) Tail current off kinetics measured on mutant receptors expressed in HEK cells. Receptors containing wild-type GluN3A subunits were treated with TCEP. N1-CS-CS refers to the GluN1 ABD C744S-C798S mutant subunit and N3-CS-CS to the GluN1 ABD C859S-C913S

mutant subunit. Mean values from the different groups are not significantly different (P=0.891, One way ANOVA on ranks, [n=4-7]).

(d) Same as in (c) but from *Xenopus* oocytes recordings, and with DTE used as a reducing agent instead of TCEP. Mean values from the different groups are not significantly different (P=0.062, One way ANOVA on ranks, [n=5-13]).

Supplementary references

1. Kvist, T., Greenwood, J. R., Hansen, K. B., Traynelis, S. F. & Brauner-Osborne, H. Structure-based discovery of antagonists for GluN3-containing N-methyl-D-aspartate receptors. *Neuropharmacology* **75**, 324-336 (2013).
